# A Pilot Study of Improving Self-Regulation and Social Interaction with Peers: An “Exciting School”

**DOI:** 10.3390/children9060829

**Published:** 2022-06-03

**Authors:** Dulce Romero-Ayuso, Beatriz Espinosa-García, Elena Gómez-Marín, Nicolás Gómez-Jara, Claudia Cuevas-Delgado, Irene Álvarez-Benítez, José-Matías Triviño-Juárez

**Affiliations:** 1 Department of Physical Therapy, Occupational Therapy Division, Faculty of Health Sciences, University of Granada, 18016 Granada, Spain; jbeatrizesp13@correo.ugr.es (B.E.-G.); gomezmarinelena@gmail.com (E.G.-M.); nicolasgomez@correo.ugr.es (N.G.-J.); claudiacuevas@correo.ugr.es (C.C.-D.); reneeab97@gmail.com (I.Á.-B.); 2 Brain, Mind and Behaviour Research Center (CIMCYC), University of Granada, Campus Universitario de Cartuja S.N., 18011 Granada, Spain; 3 Instituto de Investigación Biosanitaria ibs. GRANADA, 18012 Granada, Spain; 4 Department of Radiology and Physical Medicine, University of Granada, 18016 Granada, Spain; jmtjuarez@ugr.es

**Keywords:** social skills, occupational therapy, school, children, self-regulation

## Abstract

Social interaction skills are related to successful academic performance and mental health. One of the key elements of socio-emotional competence is self-regulation. The main aim of this study was to analyze the effect of a self-regulation program at a primary school on the social interactions of neurotypical children and children with special educational needs, from the teachers’ and parents’ perspectives. A pre-post study was conducted. The children (*n* = 107) followed 10 sessions, each one of 50 min, for ten weeks, between January and April 2021. To assess the changes in children’s social interaction, the Peer Social Maturity Scale was administered to the teachers. After the intervention, parents completed a questionnaire designed ad hoc to understand the effectiveness of children’s emotional self-regulation. The results showed a statistically significant improvement in peer interaction skills. The families were satisfied with the program, due to the improvement in their children’s knowledge about their own emotions and those of the other people, and the learning strategies to regulate their emotions. Likewise, parents indicated that it would be necessary to complement the program with teaching and emotional regulation strategies for them. The “Exciting School” program could help improve the social skills of school-aged children.

## 1. Introduction

There is an increasing interest in the development of school-based programs to improve social-emotional skills in childhood. Socio-emotional skills are understood as those that allow people to express, understand and regulate their thoughts, emotions, and behaviors in everyday situations and interactions with others, and adapt to changing conditions [1]. An adequate social adjustment requires understanding and emotional regulation [2]. Children can identify emotions and understand their causes around the age of nine, although only 60% of children at that age understand that emotions can be regulated [3].

Children with high socio-emotional competence have better social skills, more stable relationships, and better problem-solving skills. This is linked to better concentration, which affects their academic success [4] and learning [5,6]. However, few educational programs include formal or explicit training on emotional education within the curriculum [7]. This shortfall has been pointed out by different authors who are planning the specific programs to incorporate in schools [3,8], and this topic has been recognized as a challenge for occupational therapy in the school setting [9].

In this sense, the Collaborative for Academic, Social, and Emotional Learning (CASEL; https://casel.org/ accessed on 21 January 2022) promotes the adoption of policies, standards, and guidelines for the incorporation of Social and Emotional Learning (SEL) in schools [1]. CASEL defines five types of competencies for social and emotional learning that can be developed throughout life: (1) Self-awareness: the ability to identify one’s own emotions and values and understand how they guide behavior; (2) Self-regulation: the ability to control and modulate emotional expressions (positive or negative) and to interact with others in an increasingly complex way according to social rules, adapt to emotionally challenging situations, and inhibit inappropriate behaviors; (3) Social awareness: understanding social norms of behavior and being able to adopt perspectives and empathize with others; (4) Social skills: the ability to be a good listener, cooperate with others and resist negative social pressure; (5) Socio-emotional autonomy: being able to make constructive decisions about behavior based on social norms and ethical standards [10]. It is important that throughout the school years, children receive the necessary support to promote development [11]. As a consequence, different programs have been developed that include executive functions, effortful control, and socio-emotional skills, such as Second Step [5], Incredible Years Therapeutic Dinosaur Programme [12], RULER Program [13], and Smiling is Fun [14], among others. In Occupational Therapy domains, the Alert Program is frequently implemented [15], which is focused to help children understand their emotional states and improve their self-regulation in different activities and contexts. The Alert Program is one of the best-known self-regulation programs for school-age children, and it has been implemented in children with developmental disorders and in neurotypical children, although there is little evidence of its effectiveness [15,16].

Improving the social and emotional well-being of the child population in vulnerable situations is a priority objective in mental health programs. It has recently been indicated that between 10 to 20% of the world’s population of children and adolescents have mental health problems, with the main cause of disability emerging at these ages [17]. The relevance of the development of emotional well-being in the educational field has been emphasized, considering that schools or educational centers are the ideal contexts to promote their development from the beginning of formal education to university education, to improve the quality of life, prevent the onset of mental disorders and improve academic performance [8,10]. In addition, programs to improve children’s socio-emotional skills in risk contexts, and behavioral and/or self-regulation problems could prevent subsequent disorders and promote mental health in childhood [12]. Despite the availability of different programs, it is noted that a weakness of them is the lack of generalization of skills to daily living [18] or transferring skills from home to the school setting [12,18]. Despite the recognized importance of socio-emotional development and self-regulation, in our context, there are no regular programs implemented in educational centers. Interventions focused on improving self-regulation are considered transdiagnostic interventions, that promote mental health and usually are performed by professionals such as occupational therapists, which can reduce the burden on teachers [17].

Therefore, to the best knowledge of the authors, in our context (Spain), there are no programs from the Occupational Therapy approaches that try to improve self-regulation in the school setting. The “Exciting School” program arises from an educational innovation project developed through the collaboration of teachers and occupational therapists. The main aim of this study was to analyze the effect of a self-regulation program at a primary school on the social interactions of children with special educational needs and neurotypical children from the teachers’ and parents’ perspectives. The study hypothesis was that the children would improve their social-emotional skills after participating in the program.

## 2. Materials and Methods

A pre-test and post-test study were conducted with a single group.

### 2.1. Participants

Participants were children at a public primary education school. To be included, the children had to have reading and writing skills according to their educational level, aged between 7 and 10 years old, and with no diagnosis of autism spectrum disorder (ASD) moderate or severe. The initial sample consisted of 128 children between 7 and 10 years old who were recruited in coordination with teachers and psychologists, using a non-probabilistic convenience sampling procedure [17]. Written informed consent was obtained from the parents prior to the intervention. The final sample included 107 children between 7 and 10 years old (median = 8 years; interquartile range [IQR] = 8–9 years). Of the 107 children, 93.5% (*n* = 100) were neurotypical and 6.5% (*n* = 7) were children with special educational needs that had a neurodevelopmental or learning disorder (Dyslexia; Attention Deficit Hyperactivity Disorder [ADHD]; ASD; and Specific Language Disorder [SLD]) (Table 1). The diagnoses of these seven children were based on the reports by clinical psychologists and neuro-pediatricians provided by the parents to the educational and psychological orientation team of the school and they had no other comorbidities. Children with special educational needs had a curriculum adapted by the educational and psychological orientation team according to their skills, which consisted of, for example, being given extra time to finish their exams, avoiding auditive interferences during instructions, sitting close to the teacher, an emphasis on organizing and planning skills, receiving speech-language therapy, etc.

Regarding the characteristics of the sample, there were no differences in the distribution of gender (*p* = 0.566), special needs (*p* = 0.084), or disorder (*p* = 0.358) by primary school level (Table 1).

### 2.2. Instruments

The Peer Social Maturity Scale (PSMAT) was used pre- and post-intervention. It is a questionnaire for teachers that measures the social interaction skills of children and consists of 7 items. Scores on the PSMAT range from a minimum of 7 (below the score for that age) to a maximum of 49, so the higher the score the better social interaction skills [19]. The reliability of the scale is good, with a Cronbach’s alpha = 0.88. This scale is short, which makes it easy for teachers to complete it.

Likewise, an ad hoc questionnaire was designed for parents that included eight questions about the perception at home of the effectiveness of the program on the emotional self-regulation of their children. All questions could be answered with “yes” or “no”. This questionnaire was answered by 37% (*n* = 37) of the parents of neurotypical children: Nine (24.3%) were parents of children in the second year of primary school, sixteen (43.2%) were parents of children in the third year of primary school, five (13.5%) were parents of children in the fourth year of primary school, and seven (18.9%) were parents of children in the fifth year of primary school. Regarding the parents of children with special educational needs, the questionnaire was answered in all cases (100%, *n* = 7): Three (42.8%) were parents of children in the third year of primary school, two (28.6%) were parents of children in the fourth year of primary school, and two (28.6%) were parents of children in the fifth year of primary school.

### 2.3. Procedure

Approval was obtained by the Educational Community and the Ethics Committee of the University of Granada (code:1018/CEIH/2019; 13 January 2020). The program was part of a teaching innovation project of the “Parque de las Infantas” public school, of the Junta de Andalucía. Before starting the intervention, a meeting was held with all the teachers and the management team of the school to explain the procedure and the role of the teachers in each session. Likewise, a previous meeting was held with all the parents to explain the project and the objectives, requesting informed consent for their children to participate. An assessment session was held one week before the start of the intervention, in which the teachers filled in a copy of the PSMAT questionnaire corresponding to each student in their classroom. This procedure was repeated once the intervention had finished.

The “Exciting School” program was based on the concept that regulation involves behavioral, emotional, and cognitive modulation [20,21,22] and also on the theoretical framework of emotional education which includes five elements: emotional awareness, emotional regulation, emotional autonomy, social competencies, and competencies for life and well-being [7,8]. This program incorporated different activities: role-playing, stories, small discussion groups, and games designed for the interiorization of socioemotional skills. All the activities were conducted by occupational therapists, a teacher, and a neuropsychologist. Ten sessions, each one of 50 min, were held for 10 weeks, between January and April 2021, in the tutorial plan schedule (Table 2). All the teachers responsible for each classroom remained and participated while the activities were conducted, together with the occupational therapist. The sessions had the same structure: remembering and reviewing the contents of the previous session, performing the main activity of the session, and closing it with an interactive quiz, called Kahoot! which reviewed the knowledge we had experienced during the session.

Once each session ended, the teacher sent all parents a summary video about the weekly intervention of no more than 3 min through a WhatsApp group for each course. In each session, the children were asked to do a weekly task at home according to the planned objectives, which was useful to consolidate their skills (Table 2). In addition, the information was included in the school blog so that all parents and teachers could access it anytime they wanted (https://colegioparquedelasinfantas.blogspot.com/2021/04/emociones-ultimas-sesiones.html accessed on 12 May 2021).

### 2.4. Data Analysis

To examine data normality, the Kolmogorov-Smirnov test (if *n* > 50) or Shapiro -Wilk test (if *n* ≤ 50) were used. The normality test of the data showed that they did not follow a normal distribution. Thus, median and IQR were used for their description. Categorical data were given as absolute frequencies and percentages. The chi-square test (or Fisher’s exact test in case of the expected values in any of the cells of the contingency table were below five) were performed to analyze differences between proportions. To analyze the pre-post intervention differences, the Wilcoxon signed-rank test was used. For a better understanding of the pre-post intervention differences, together with the median and IQR, mean and standard deviation (SD) have been included in the Results section. The significance level for all tests was set at *p*  <  0.05. Statistical analysis was performed using the IBM Statistical Package for Social Sciences Software (IBM Corp. Released 2012. IBM SPSS Statistics for Windows, Version 25.0, Armonk, NY, USA).

## 3. Results

### 3.1. Pre-Post Intervention Differences in PSMAT Scoring in Neurotypical Children

Of the 100 children included in the neurotypical group, two left for other schools, so the results refer to the remaining 98 children who did complete the study. After the children participated in the program, statistically significant changes were observed in: “skills for appropriately standing up for own opinions, needs and rights with peers”, *p* < 0.001; “skills for joining new groups of peers, or welcoming a new child into the group”, *p* < 0.001; “skills for coping with peers who frustrate or interfere with the group’s goals and activities”, *p* < 0.001; “skills for understanding the needs and interests of peers who differ from the group norm”, *p* < 0.001; “maturity of the child’s everyday modes of playing with peers”, *p* < 0.023; and “PSMAT total score”, *p* < 0.001 (Table 3).

### 3.2. Pre-Postintervention Differences in PSMAT Scoring in Children with Special Educational Needs

All children with special educational needs (*n* = 7) completed the study and their results showed that after the intervention, there were no statistically significant changes found in any of the items nor the PSMAT total score (Table 3).

### 3.3. Parents’ Perception of Neurotypical Children of the Effectiveness of the “Exciting School” Program

A higher proportion of parents reported that the program influenced their children’s daily lives. Additionally, a higher proportion of children explained to their parents the self-regulation strategies they learned. Most of the parents indicated that their children used self-regulation strategies at home after the program. Likewise, most of the children understood their emotions better and recognized better the emotions of others after the program. Furthermore, a higher proportion of parents thought that their children had better self-regulation after the intervention. Parents considered it necessary to learn self-regulation strategies. Finally, most parents found useful the information provided to them weekly through the school blog and from the responsible parents and teachers in each group (Table 4).

### 3.4. Parents’ Perception of Children with Special Educational Needs of the Effectiveness of the “Exciting School” Program

All parents thought that the program influenced their children’s daily lives. Most of the parents reported that their children explained to them the self-regulation strategies learned. A higher proportion said that their children understood better their own emotions and recognized more effectively the emotions of others. Almost all parents thought that they needed to learn self-regulation strategies. All parents found useful the information we provided them weekly through the school blog and from the responsible parents and teachers in each group. Less than half of the parents found that their children used self-regulation strategies at home and that they had better self-regulation (Table 4).

## 4. Discussion

The main aim of this study was to analyze the effect of a self-regulation program at a primary school on the social interactions of children with special educational needs and neurotypical children from the teachers’ and parents’ perspectives. This objective has become increasingly important in referrals made to occupational therapists in recent years [23]. In fact, some studies have indicated that children with low social skills showed difficulties in emotional regulation, and this factor should be considered in interventions performed by occupational therapists [9].

The preliminary findings of this pilot study have found that emotional self-regulation skills, assertiveness, empathy, and skills to initiate conversation and social interactions improved in neurotypical children, but not in children with special educational needs. According to the CASEL Social-Emotional Learning Framework, neurotypical children increased emotional self-awareness, self-control, responsible decision-making, social awareness, and social skills [10]. These changes were reported by teachers and parents, which could suggest that there was a transfer of learning to the family environment. Similar results have been observed when learning environments provide positive affective relationships between students and teachers, especially when teachers encourage children to develop their own opinions, making their learning more meaningful, thus creating inclusive classrooms [13,24,25,26,27]. Teachers are privileged observers when assessing children’s interaction skills [9]. They can help to have a more optimistic view of students, facilitating the teaching-learning process [26]. Likewise, the results of our program are consistent with the proposals that suggest that an optimal level of classroom structure and control allows the development of higher levels of social maturity and independence [11]. One of the reasons why our program might show positive results is because it includes the dimensions indicated by other authors for the effectiveness of self-regulation programs in schools [11,28]. Our program incorporated explicit teaching of self-regulation skills through activities, mainly with play, that was conducted routinely and organized on a specific schedule with weekly feedback from occupational therapists. Additionally, the program included the SAFE criteria: (S) sequential activities for the development of skills; (A) active participation of children; (F) focused time for the practice of socio-emotional skills, and; (E) explicit definition of the skills we developed in each session [11]. Additionally, our results coincide with other studies with preschoolers that also implement cognitive and emotional self-regulation in the intervention [28] and indicate that after intervention programs, emotional understanding and social competence were improved [16,29]. Similar to our results, other occupational therapy self-regulation programs, such as the Alert program, have shown improvements in socio-emotional competencies in children between three and six years old [25] and in adolescents between 12–13 years old [30]. Moreover, developing emotional autonomy and social skills can help them resolve interpersonal conflicts and facilitate their participation in the school [9] as shown by the results of the maturity of the child’s everyday modes of playing with peers in this study.

In the case of children with special educational needs, it is possible that the lack of significant differences is due to the small number of children in our sample. It could also be because the strategies used are generic and some of these children need a specific therapeutic intervention for the recognition of emotions, social cognition, and putting oneself in another’s place, etc., due to deficiencies in these surroundings, as occurs in children on the ASD [31,32,33]. Likewise, in children with ADHD, difficulties in self-regulation and high emotional reactivity have been observed. In addition to the strategies used, these children require more specific therapeutic intervention to improve the cognitive reevaluation of mood enhancement or the use of distraction [34].

Involving parents in self-regulation improvement programs could improve the effectiveness of the program and the transfer of socioemotional skills to other contexts, such as the playground or at home. Social-emotional learning programs have greater effects when families are involved in the program and are part of it. Parents participated in our program through the school blog, in an informative way, and they were asked for their involvement with the homework, with the intention that the children could extend what they learned in each weekly session to daily life [16]. Our results support the recommendations made to improve self-regulation programs, such as the Alert Program, with the incorporation of parents in programs focused on socioemotional interventions [16,35]. Regarding the differences found in the parents’ perception of the effectiveness of the program, it should be noted that most parents indicated that the intervention had a positive influence on the children’s daily life. In both groups, parents reported that their children recognized better their own emotions and the emotions of others. Although both groups positively valued the information provided by the school blog and through the tutors, they considered that a workshop for parents would be necessary to improve their children’s self-regulation strategies. These could be explained because parents are concerned about the socio-emotional development of their children and consider it a topic of interest. It is possible that these findings may be related to the COVID-19 pandemic, where parents have spent more time with their children and prioritized emotional well-being over other goals such as academic achievement [36]. Specifically, it has also been observed that during the COVID-19 period, parents spent more time helping their children learn. This increase in the presence and emotional and temporary availability of parents has been related to an increase in children of positive emotions, and prosocial behavior and a decrease in externalization and internalization of disruptive behaviors [36]. Although our study was conducted after the confinement period of COVID-19, sanitary measures required the use of a mask. This fact could have influenced the results obtained in the group of children with special educational needs, by making it more difficult for them to recognize emotions due to the use of masks [36]. Similar to the information reported by the teachers, parents of children with special educational needs indicated that they did not use self-regulation strategies at home in a spontaneous way, and they kept showing a reduced awareness of their own emotions. These results suggest that although the program has shown positive results, children with special educational needs could benefit from a more specific therapeutic intervention that complements the “Exciting school” program.

This study has several limitations. First, the sampling was intentional. Second, due to this study being a pilot one, it might limit the possibility of formally examining and interpreting mediation and change mechanisms implicated in the results found. Third, we did not collect information about the children’s socioeconomic status. It would be recommended that future studies include this variable to determine its relationship with the effects of interventions aimed to improve self-regulation in childhood. Fourth, less than half of the parents of neurotypical children and all the parents of children with special educational needs answered the questionnaire about satisfaction with the “Exciting School” program. Therefore, the results might have shown an overestimation of the perception of the effectiveness of the program because the parents who have responded could be the most motivated and with higher expectations about the benefits of this intervention.

Our study might contribute to improving the evidence of the presence of occupational therapy in the school setting through a self-regulation program. Further longitudinal studies are needed to know the effectiveness of the “Exciting School” program, which incorporates direct assessments from children allowing us to verify whether it increases the emotional well-being of the child and makes them more effective in facing problems and making decisions. Likewise, it would be recommendable for future studies to have a comparison control group.

The preliminary findings of this study show the usefulness of programs that promote emotional well-being and socio-emotional skills in the school context, from an inclusive and transdiagnostic perspective, that improve the recognition of emotions in themselves and in others and learn self-regulation strategies. Collaboration of teachers, parents and occupational therapists is essential for the learning, implementation and development of social-emotional skills in the school population.

## 5. Conclusions

The inclusion of social-emotional development and self-regulation programs in the academic curriculum with active and play-based methodologies could contribute to establishing a comprehensive climate where everyone learns to accept differences and diversity. Finally, parents consider this type of program essential and would like to receive more information and training from the school.

## Figures and Tables

**Table 1:** Characteristics of children by primary school level (*n* = 107).

	Second *n* (%)	Third *n* (%)	Fourth *n* (%)	Fifth *n* (%)	*p* Value
**Neurotypical (*n* = 100)**					
Gender					0.538 *
Boys	13 (59.1%)	17 (41.5%)	11 (50%)	6 (40%)	
Girls	9 (40.9 %)	24 (58.5%)	11 (50%)	9 (60%)	
**Special Educational Needs (*n* = 7)**					
Gender					0.084 **
Boys	0 (0%)	3 (100%)	1 (50%)	0 (0%)	
Girls	0 (0%)	0 (0%)	1 (50%)	2 (100%)	
Disorder					0.358 **
Dyslexia	0 (0%)	2 (4.3%)	1 (4.2%)	0 (0%)	
ADHD	0 (0%)	1 (2.1%)	0 (0%)	0 (0%)	
ASD	0 (0%)	0 (0%)	1 (4.2%)	1 (3.8%)	
SLD	0 (0%)	0 (0%)	0 (0%)	1 (3.8%)	

*: Chi-square test; **: Fisher’s exact test; ADHD: attention deficit hyperactivity disorder; ASD: autism spectrum disorder; SLD: specific language disorder.

**Table 2:** Summary of “Exciting School” program.

Domain	Aims	Activities
Emotional Recognition, Comprehension, and Expression	To recognize one’s own simple emotions To recognize simple interpersonal emotions To recognize the causes of emotions To acquire and use vocabulary to describe emotions To recognize own and other complex emotions To understand the causes of complex emotions To understand the causes of emotional ambivalence situations	SESSION 1: The Parcheesi of emotions. Kahoot! Homework: The diary of emotions and Body Scanner. SESSION 2: In my neighbor’s shoes. Kahoot! Homework: Little Sherlock: Investigating the emotions around me. SESSION 3: Let’s play: Guess how I feel in my daily life. Kahoot! Homework: How I feel, how I do?
Emotional Self-regulation	To learn basic sensory strategies for self-regulation To learn complex strategies for self-regulation To apply techniques to change the negative emotional state	SESSION 4: Move and keep calm! Kahoot! Homework: my sensory world and my calm: The bottle of calm. SESSION 5: The traffic light and Spaghetti techniques. Kahoot! Homework: Practice spaghetti with the traffic light in my daily life. The diary of anger. SESSION 6: The tale of the turtle. Kahoot! Homework: The Turtle Bookmark. SESSION 7: Collage of coping strategies. Kahoot! Homework: What works for me?
Emotional Autonomy and Social Skills	To promote basic social skills To demonstrate respect for others To increase a positive attitude and personal self-efficacy To enhance self-esteem	SESSION 7: I Liked myself! Positive Self-Talk. Kahoot! Homework: Creating my healthy habits roulette! SESSION 8: The invisible backpack: stones or bubbles? Kahoot! Homework: Practice being nice. How can I help you? SESSION 9: Use of messages “I feel …” Improving our communication. Kahoot! Homework: Letter of Thanks.
Life-skills and Well-Being	To promote occupational balance To learn skills of management of time	SESSION 10: The time thief. Kahoot! Homework: Occupational balance in my life!

**Table 3:** Peer Social Maturity Scale (PSMAT) Items. Pre-post intervention differences in the “Exciting School” program in neurotypical children and children with special educational needs.

	**Neurotypical** (*n* = 98)				**Special Educational Needs (*n* = 7)**			
	**Pre-Intervention**	**Post-Intervention**			**Pre-Intervention**	**Post-Intervention**		
	**Median (IQR)** Mean (SD)	**Median (IQR)** Mean (SD)	**Z**	***p-*** **Value ^a^**	**Median (IQR)** Mean (SD)	**Median (IQR)** Mean (SD)	**Z**	***p-*** **Value ^a^**
Skills for appropriately standing up for own opinions, needs, and rights with peers	5 (4–6) 4.63 (1.37)	5 (4–6) 5.13 (1.25)	−5.21	<0.001	2 (1–3) 2.57 (1.72)	3 (2–4) 3 (1.63)	−1.73	0.083
Skills for joining new groups of peers, or welcoming a new child into the group	5 (4–6) 4.83 (1.33)	5 (4–6) 5.23 (1.28)	−3.71	<0.001	2 (2–4) 2.86 (1.68)	3 (2–5) 3 (1.53)	−0.58	0.564
Child’s leadership skills with peers	4 (3–6) 4.39 (1.52)	5 (4–5) 4.55 (1.39)	−1.56	0.119	2(1–3) 2.29 (1.38)	2 (1–4) 2.43 (1.62)	0	1
Skills for coping with peers who frustrate or interfere with the group’s goals and activities	4 (3–6) 4.49 (1.55)	5 (4–6) 4.88 (1.36)	−3.57	<0.001	2 (1–3) 2.57 (1.72)	3 (1–3) 2.43 (1.51)	−0.58	0.564
Skills for understanding the needs and interests of peers who differ from the group norm	4 (3–6) 4.49 (1.49)	5 (4–6) 4.88 (1.36)	−3.50	<0.001	2 (1–3) 2.57 (1.72)	3 (1–3) 2.43 (1.51)	−0.58	0.564
Maturity of the child’s everyday modes of playing with peers	5 (4–6) 4.98 (1.41)	5 (4–6) 5.22 (1.34)	−2.28	0.023	3 (2–4) 3 (1.29)	3 (2–4) 2.86 (1.35)	−0.45	0.655
The overall maturity of the child’s social skills	5 (4–6) 4.85 (1.31)	5 (4–6) 4.95 (1.27)	−1.21	0.228	3 (2–3) 2.86 (1.57)	3 (2–4) 2.86 (1.35)	0	1
PSMAT total score	32 (27–40) 32.66 (8.41)	35 (29–42) 34.85 (8.34)	−4.29	<0.001	17 (13–21) 18.71 (10.37)	20 (11–28) 19 (9.97)	−0.11	0.917

IQR: interquartile range; SD: standard deviation; ^a^: Wilcoxon signed-rank test; PSMAT: Peer Social Maturity Scale.

**Table 4:** Parent’s perception of the effectiveness of the “Exciting School” program (*n* = 44).

	Neurotypical (*n* = 37)	Special Educational Needs (*n* = 7)
	*n* (%)	*n* (%)
Influence of Program in Daily Life		
Yes	23 (62.2%)	7 (100%)
No	14 (37.8%)	0 (0%)
Explained strategies of self-regulation to parents		
Yes	33 (89.2%)	6 (85.7%)
No	4 (10.8%)	1 (14.3%)
Use of strategies of self-regulation at home		
Yes	23 (62.2 %)	2 (28.6%)
No	14 (37.8%)	5 (71.4%)
Understand better their emotions after the program		
Yes	34 (91.9%)	4 (57.1%)
No	3 (8.1%)	3 (42.9%)
Better recognition of the emotions of others		
Yes	32 (86.5%)	5 (71.4%)
No	5 (13.5%)	2 (28.6%)
Better emotional self-regulation		
Yes	28 (75.7%)	3 (42.9%)
No	9 (24.3%)	4 (57.1%)
Parents think they need a program to learn self-regulation strategies		
Yes	36 (97.3%)	6 (85.7%)
No	1 (2.7%)	1 (14.3%)
Usefulness of information provided through the school blog		
Yes	33 (89.2%)	7 (100%)
No	4 (10.8%)	0 (0%)

## Data Availability

The data are available from the corresponding author upon request.
